# A Christian City

**Published:** 1890-05-31

**Authors:** 


### The Hospital

A Weekly Journal of

Science, Medicine, Nursing, and Philanthropy.

*For Hospitals, Asylums, Medical Practitioners, Students, Nurses, Families, Parishes, Congregations, and the Charitable Public.*

Vol. VIII.—No. 192.] [May 31, 1890.

### A CHRISTIAN CITY.

A Christian City, like any other collection of human beings, has its distinguishing marks and its characteristic periodical movements. There is a life and activity of the whole body corporate, as there is of the individual parts; and just as a man gradually makes clear his character to his neighbours by his sayings and doings, so does a city in course of time establish a reputation in the eyes of the country and of the world. London is distinctly a Christian City, as ancient Rome was distinctly and essentially Heathen. A considerable minority of educated men, and possibly a majority of the lowest classes, hold that Heathenism is preferable to Christianity. They think that the latter imposes unscientific limits upon their freedom of thought and action. The more prominent features of the life of ancient Rome are quite familiar to ordinary persons, and may properly be compared with the most marked characteristics of the London of to-day. Such a comparison, if fairly and intelligently made, cannot but be both interesting and serviceable.

Those cultured men who hanker after what they consider the larger intellectual and physical freedom of heathen times and peoples will not allow it to be taken for granted that there was less actual public and private virtue in ancient Rome than there is in London to-day. Rome had her ways; we have ours. Our ways are different from hers, it is said; but they are not essentially better. But we affirm that our ways are in themselves and of their own essence more publicly and privately useful and more endowed and charged with moral excellence than were the ways of ancient Rome. Take for example a Roman Triumph! We will put ourselves in the place of the hero of the hour. He has been commissioned by the State to command one of her armies in the field; he has met and vanquished her enemies, not once, nor twice, nor thrice, but many times; he has finally, on some supreme occasion, faced and overthrown a more than usually dangerous foe; he has taken captive the leader of the enemy with the flower of his army. He returns to the Imperial City to receive the just reward of his deeds. A Triumph is decreed to him. What such a Triumph means to the victorious leader every student knows, and he knows also what it means to the captives. Led like slaves at the car of the conqueror they slowly march to the destined spot, and then are told off for death.

Now, putting ourselves in the place of the victor, we see nothing necessarily immoral in the Triumph that has been decreed to him, nor in the sentiments of pride that animate his bosom on the occasion He is a man; he has proved himself a hero; and his country gives him but his just due when it acclaims him defender and saviour of the State. But who so much as thinks upon the doomed victims at his chariot wheels? True they have but experienced the common fortunes of war. Their hearts may be swelling at the very moment with as noble a scorn of defeat as the conqueror's pride of victory. They know well that had fate been more propitious they would have been seated on the triumphal car, and the proud victor might have been dragged at its wheels. But, we ask, where is the public utility of the butchery of those brave captives; and still more, where is the moral excellence? The war is over; the enemy is defeated; the danger to the State is passed; more than enough blood has been shed; the time for a gracious clemency has arrived. We have not selected, as is commonly done, the arena, where gladiatorial shows and conflicts between despairing men and savage wild beasts gave zest and piquancy to the enjoyment of the holiday crowds. In order to do the fullest justice to the character of the life of ancient Rome, we have chosen for comparison the altogether nobler circumstance of the Triumph of the victor returning from hard-fought fields.

Christianity has introduced a totally different way of thinking, and new standards of public and private judgment. The defender of the State has done nobly in that he fought for his country. But where is the nobility of intoxicating himself with pride thereafter, and of glutting his insatiate greed of praise with the blood of his brave but defenceless enemies? Christianity has taught us that such intoxication of pride and greed of public praise is not noble, but showman-like and vulgar. We have the sense to see that Christianity is right. What a glorious magnanimity is there in this: "Love your enemies; do good to them that hate you?" The Romans did not hate the poor and the conquered; they despised them too profoundly for that. But is it philosophical; is it publicly useful to despise the poor and to let them sink to the lowest levels of vice and stupidity? It is no more philosophical and no more publicly useful than it would be for a great State to allow its land to go out of cultivation, its armies to be demoralised, or its ships to rot at sea.

In the Christian City of London on Hospital Sunday, a spectacle will be witnessed which may properly be compared with the spectacle of the Roman Triumph. In the pitch of its enthusiasm, in the universal spontaneity of sympathetic movement, Hospital Sunday will not compare with the Roman spectacle. But in the innate excellence of the inspiring motive, and in the philosophic reasonableness and public utility of the objects sought, Hospital Sunday is as much in advance of the Roman Triumph as the cultivated Englishman of to-day is of the barbarous Briton who resisted the Roman arms.

Hospital Sunday is one of our annual spectacles; it is, or should be, a Christian holiday: a triumph of the year. The preacher, above all, should feel it to be a high occasion, because it is the day when the faith he preaches proclaims itself in deeds, not words. A poor captain is he if he wears his uniform with a cold heart, and takes his part in the spectacle without fervour and without resolution. The very private soldiers of tyrant Rome, as they marched with eagles held aloft and banners floating in the breeze, to their ruthless work of enslaving or destroying the nations, might make the leaders of the Christian cause for ever ashamed if they cannot, on a day of battle, fight with the valour and self-abandoning enthusiasm of Christian heroes. Great and noble as Christianity is in itself, the spirit of its teachers is sometimes the meanest of the mean and the poorest of the poor. But for one day in the year nothing is asked for Church, nothing for minister, nothing for missions, nothing for self. The Christianity of London, under the leadership of its priests and teachers, rises up to do a gracious deed of healing to the poor sick. What a noble spectacle that deed might be! What a heroic moment it might annually mark in the triumphant progress of the universal faith! How impressive it might appear to the world: how blessed to the Church. If but the leaders of the Christian people of this city would rise to the nobleness of the occasion, Hospital Sunday would soon become one of the most conspicuous and glorious monuments of the faith of the nineteenth century.

